# Linking two DNA duplexes with a rigid linker for DNA nanotechnology

**DOI:** 10.1093/nar/gkv662

**Published:** 2015-06-30

**Authors:** Ryu Tashiro, Masahiro Iwamoto, Hironobu Morinaga, Tomoko Emura, Kumi Hidaka, Masayuki Endo, Hiroshi Sugiyama

**Affiliations:** 1 Faculty of Pharmaceutical Sciences, Suzuka University of Medical Science, 3500–3 Minamitamagaki-cho, Suzuka-shi, Mie 513–8670, Japan; 2 Department of Chemistry, Graduate School of Science, Kyoto University, Kitashirakawa-Oiwakecho, Sakyo-ku, Kyoto, 606–8502, Japan; 3 Institute for Integrated Cell-Material Sciences (WPI-iCeMS), Kyoto University, Yoshida-ushinomiyacho, Sakyo-ku, Kyoto, 606–8501, Japan

## Abstract

DNA has recently emerged as a promising material for the construction of nanosized architectures. Chemically modified DNA has been suggested to be an important component of such architectural building blocks. We have designed and synthesized a novel H-shaped DNA oligonucleotide dimer that is cross-linked with a structurally rigid linker composed of phenylene and ethynylene groups. A rotatable DNA unit was constructed through the self-assembly of this H-shaped DNA component and two complementary DNA oligonucleotides. In addition to the rotatable unit, a locked DNA unit containing two H-shaped DNA components was also constructed. As an example of an extended locked structure, a hexagonal DNA origami dimer and oligomer were constructed by using H-shaped DNA as linkers.

## INTRODUCTION

DNA is a powerful tool that can be used to create nanosized architectures and devices because of its synthetic accessibility and ability to self-assemble (1–6). Structural rigidity is important for constructing well-ordered architectures, and rigid DNA motifs based on Holliday junctions have been developed for DNA tiling and DNA origami (1,7,8). In the case of DNA machinery systems, a single-stranded DNA linker is generally used as a movable segment to connect rigid segments in the machines (9). However, these rigid segments can move randomly in any direction because of the flexible nature of the single-strand connector, which is far from an ideal motion for the machines. In this context, the concept of rigidity may be included in the design of movable segments in the DNA machine to allow more precise and higher ordered machinery motion.

**Figure 1.**
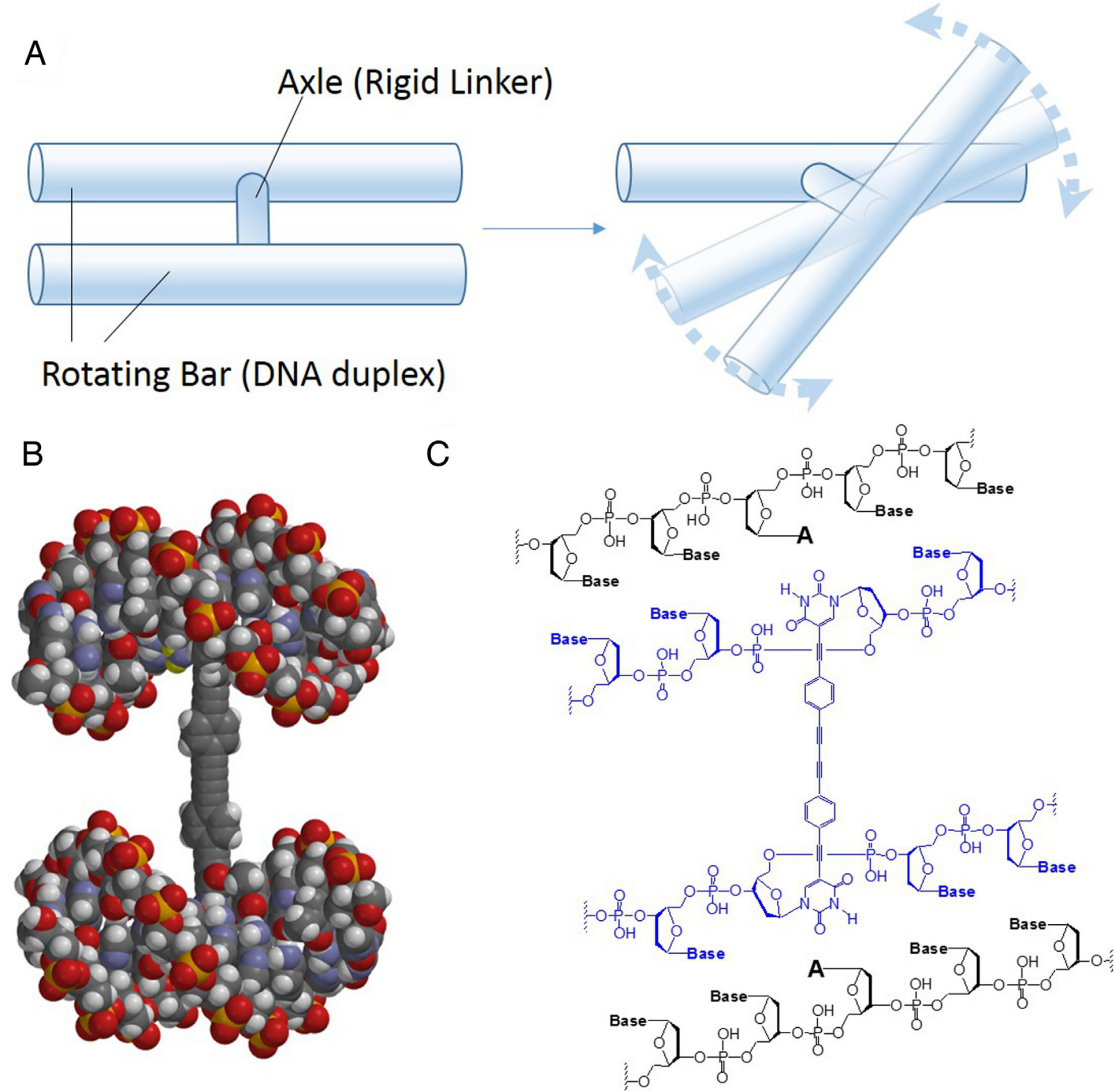
Simplified illustration (**A**), molecular model (**B**) and chemical structure (**C**) of the **RDU**.

Molecular rotators, rotors and motors are interesting machinery systems in supramolecular chemistry (10). In the field of DNA nanotechnology, however, there are no DNA-based rotary systems because of the lack of a suitable rotatable motif. A rotary unit requires a rigid linkage that connects two rotatable bars and acts as an axle to avoid contact between the two bars and allow smooth rotation. The Holliday junction motif is not useful as a rotary unit. We anticipated that exploitation of a DNA unit composed of two DNA duplexes connected by a rigid linkage acting as an axle would constitute an important first step in the construction of such DNA-based rotating systems. In this study, we designed and synthesized a DNA analogue that was capable of connecting two distinct DNA oligonucleotides without a flexible region. By using this DNA analogue, a novel rotatable DNA unit (**RDU**) comprising two DNA duplexes and a rigid linker was constructed (Figure 1).

## MATERIALS AND METHODS

### General

Chemical reagents and solvents, except for DNA solid phase synthesis, were purchased from commercial supplier (Aldrich or Wako or TCI) and used without further purification. Reagents for DNA solid phase synthesis were purchased from GlenResearch, except for FAM and Dabcyl amidite, which for ChemGenes. All the chemically modified DNA oligomers were synthesized on 1 μmol scale with an AppliedBiosystems 3400 DNA synthesizer. A standard program with 3′ CE (β-cyanoethyl) nucleotide phosphoramidites was used. All the staple DNAs for the DNA frame were purchased from Operon Biotechnology. Single-stranded M13mp18 viral DNA was purchased from New England Biolabs, Inc. Nuclear magnetic resonance spectra were recorded on a Varian VNMRS 400 or 600 MHz spectrometer. Mass spectra were recorded on a BrukerDaltonics MicroTOF-Q. Reverse-phase HPLC were performed on a Shimadzu Prominence HPLC system equipped with a Chemcobond 5-ODS-H C18 column (4.6 × 15 cm) using a SPD-M20A diode array detector at 254 nm. UV experiments were recorded on Jasco V-630BIO instrument equipped with a microcell holder. Fluorescence experiments were recorded on Jasco FP-8500 instrument equipped with a thermostatted cell holder.

### Polyacrylamide gel electrophoresis analysis

Polyacrylamide gel electrophoresis (PAGE) was carried out using 12.5% cross-linked slab gels (1 mm thick) with the following running buffer: 1 × TBE, 100 mM NaCl, 10 mM MgCl_2_. Bromophenol blue was used as tracking dye. DNA bands were visualized under LED blue light (NihonEido).

### Preparation of hexagonal DNA origami units

The design of the hexagonal DNA origami was previously reported (11). The hexagon origami was assembled in 20 μl of solution containing 10 nM M13mp18 single-stranded DNA, 50 nM staple oligonucleotides (5 equiv), 20 mM Tris buffer (pH 7.6), 10 mM MgCl_2_ and 1 mM EDTA. The mixture was annealed by heating and reducing the temperature from 85 to 15°C at a rate of −1.0°C/min. The origami solution was purified using Sephacryl S-300 gel-filtration column after finishing the annealing.

### Self-assembly of DNA origami using H-shaped DNA

For assembling the hexagonal DNA origami, two hexagonal origami monomers, **Hex 1** and **Hex 2** (5 nM), with four connection strands at the D- and A-edges, respectively, were annealed with H-shaped DNA (**H4** linker, 6–8 equiv) from 40 to 15°C at a rate of −1.0°C/min. In the case of the linear oligomeric assembly, **Hex 3** monomer (5 nM) with connectors at the A- and D-edges was annealed with **H4** linker using the same condition. The origami assemblies were directly observed by atomic force microscopy (AFM).

### AFM Imaging

AFM images were obtained using a high-speed AFM system (Nano Live Vision, RIBM, Tsukuba, Japan) with a silicon nitride cantilever (resonant frequency = 1.0−2.0 MHz, spring constant = 0.1−0.3 N/m, EBD Tip radius <15 nm, Olympus BLAC10EGS-A2). The sample (2 μl) was adsorbed on a freshly cleaved mica plate (Φ 1.5 mm, RIBM Co. Ltd., Tsukuba, Japan) for 5 min at room temperature and then washed with the same buffer solution three times. The samples were observed in the same buffer solution by high-speed AFM using tapping mode.

## RESULTS AND DISCUSSION

### Design of H-shaped DNA systems

**Figure 2.**
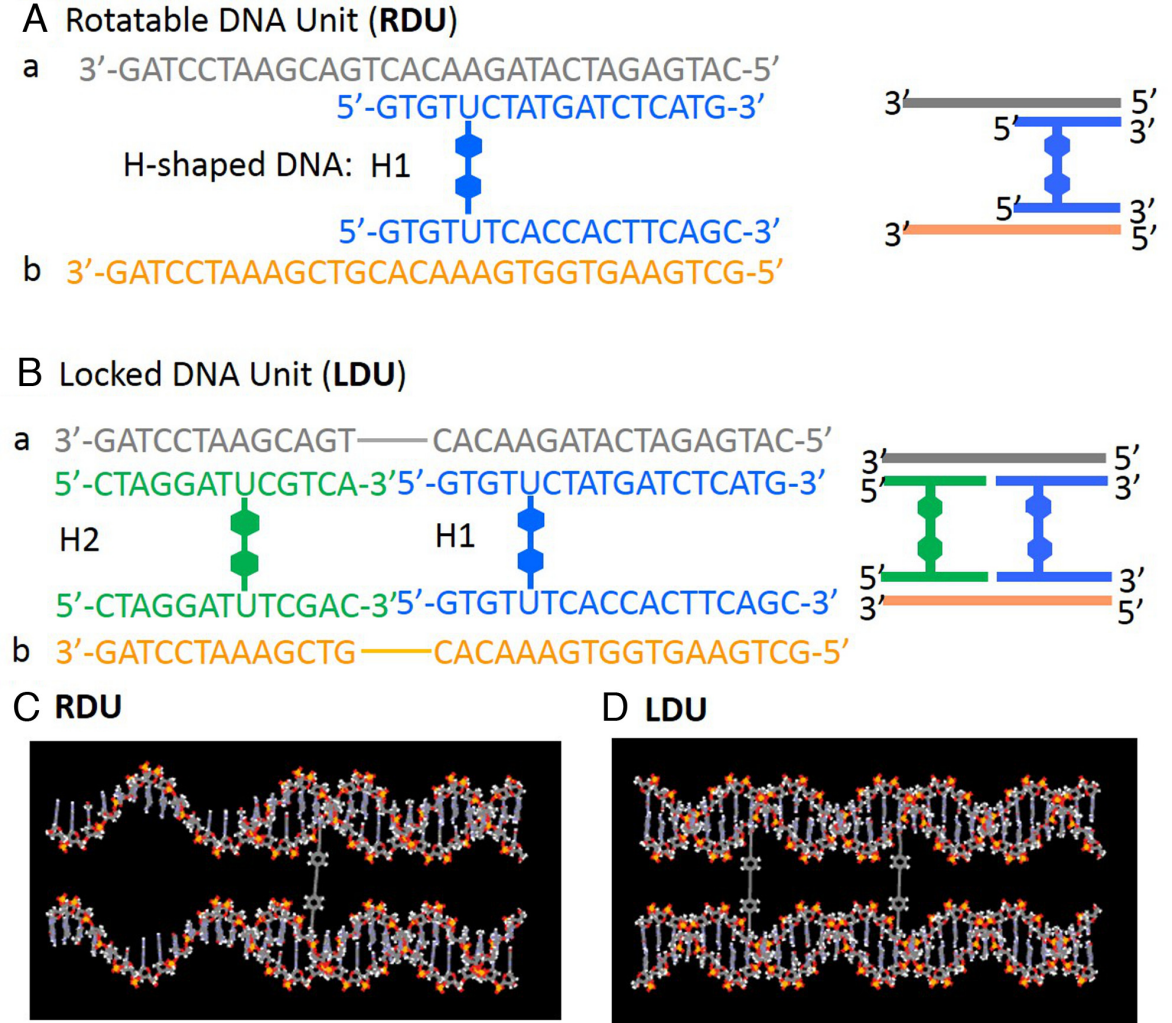
DNA sequence and molecular model of **RDU** (**A**) and (**C**), and **LDU** (**B**) and (**D**). HPLC profiles of reaction mixture from synthesis of **H1**.

To date, various types of DNA–rigid-molecule conjugates have been reported (12–17). However, in these cases, the rigid linker is connected to the flexible phosphodiester backbone of the DNA oligomer. Therefore, these connections are not suitable for our purpose. In our DNA unit design, each terminal of the rigid molecule, which is composed of phenylene and ethynylene groups, is joined through a triple bond at the 5′-position of the uridine base located in the middle of the DNA duplexes (Figure 1B). The rigid linker and the DNA duplexes serve as the axis and the rotating bars, respectively. Construction of the **RDU** required the use of a rigid linker composed of modified DNA. Therefore, our first goal was to synthesize a H-shaped DNA molecule that could be used as a building block for the **RDU**. The strategy used to form the building blocks is illustrated in Figure 2. The **RDU** was composed of a H-shaped DNA (**H1**) and two complementary DNA oligonucleotides, **a** and **b**, as illustrated in Figure 2. In addition, we designed a locked DNA unit (**LDU**) motif that was composed of two different H-shaped DNA units (**H1** and **H2**), and **a** and **b**, for comparison with the **RDU**.

### Synthesis of H-shaped DNA systems

**Scheme 1.**
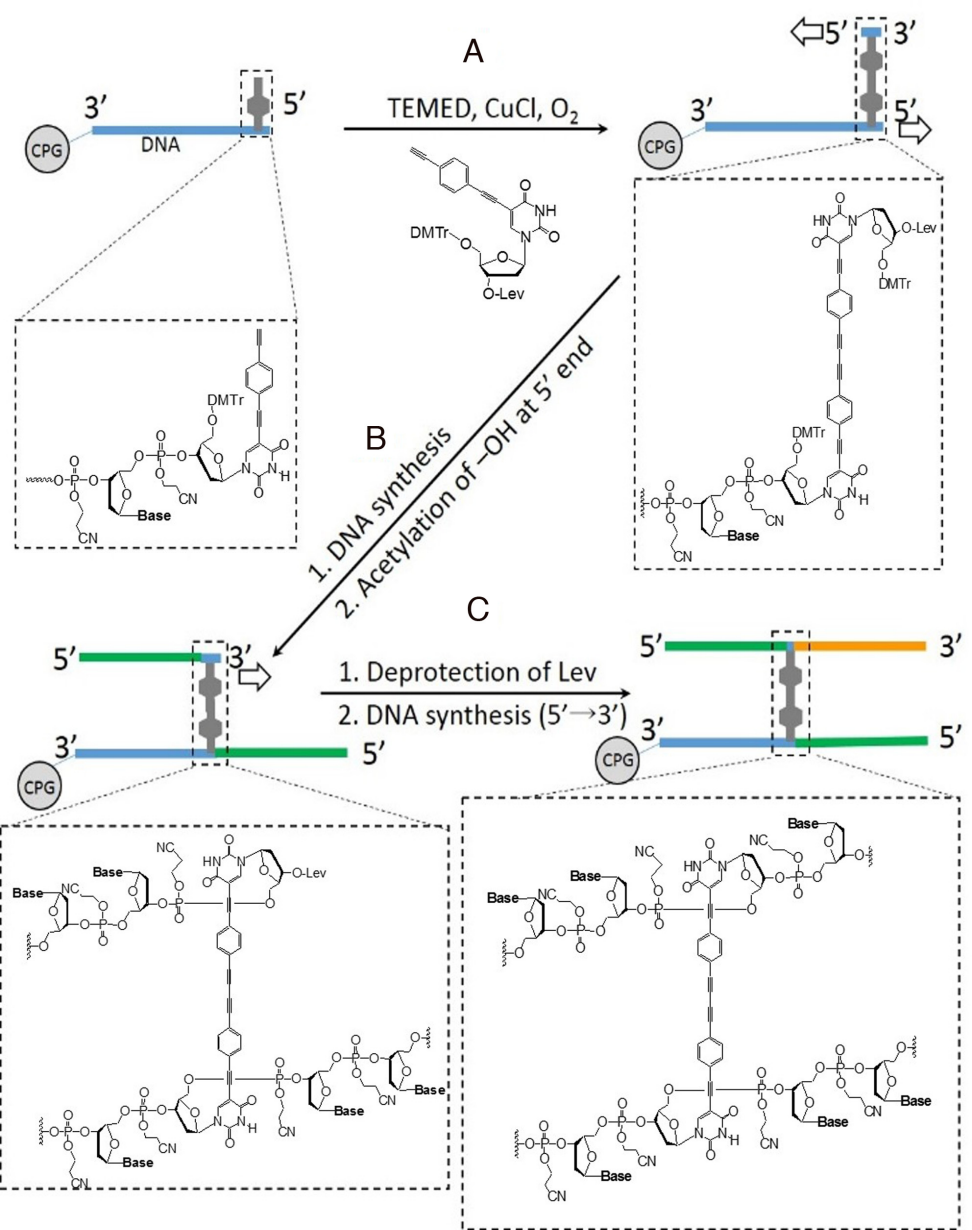
On-support synthesis of the H-shaped DNA system.

**Scheme 2.**
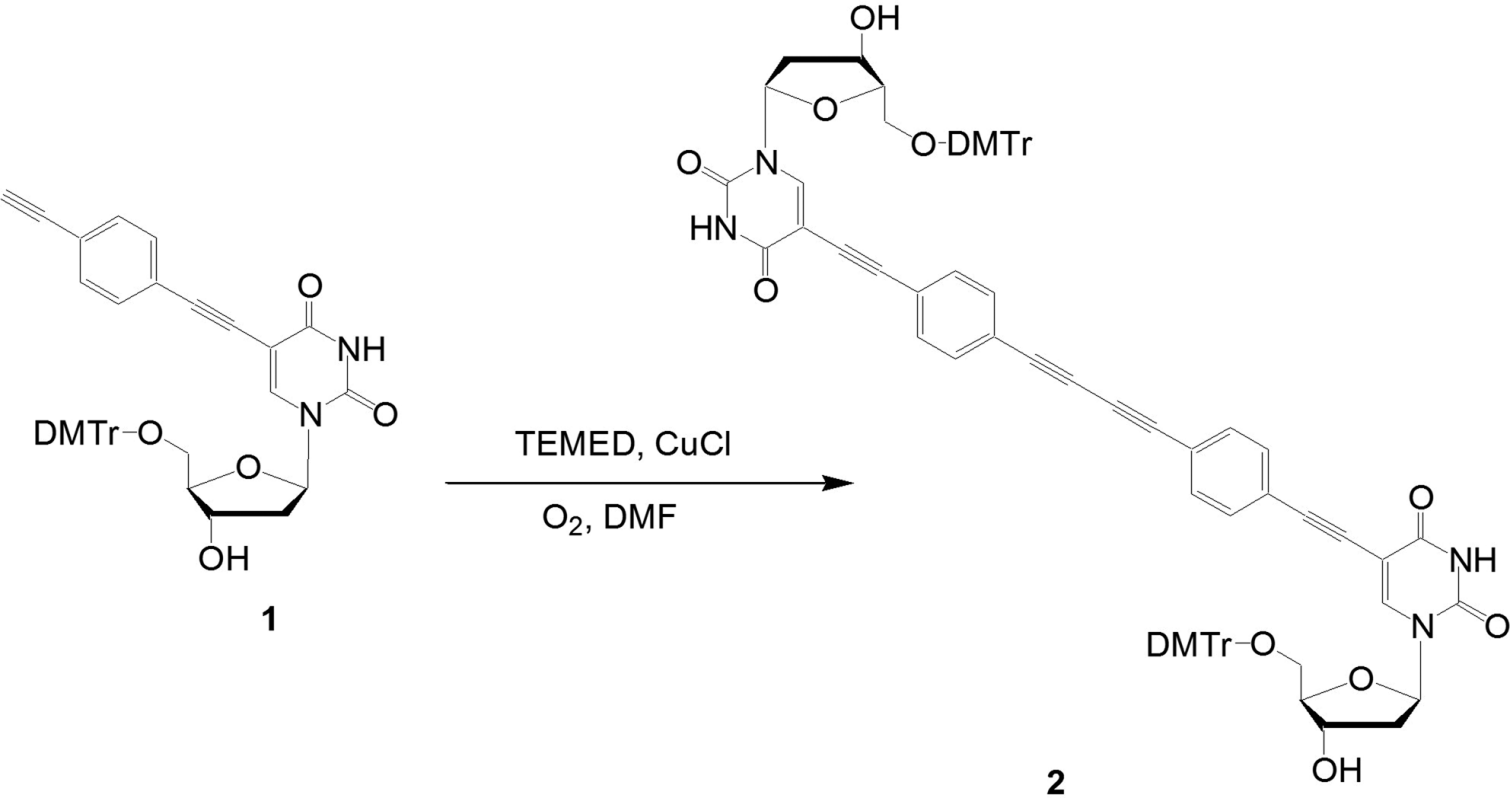
Synthesis of dimeric nucleoside **2**.

Our synthetic strategy is outlined in Scheme 1 (a more detailed scheme is shown in the Supplementary Data). The Glaser reaction is a copper-catalyzed oxidative acetylenic coupling reaction that has been used to conjugate DNA and a dye on a support (18). We utilized this on-support Glaser reaction to fabricate the rigid linkage, and used the coupled moiety as a branching point for DNA strand elongation. To our knowledge, this combination of coupling followed by elongation for branch formation is unique. Before the solid-phase reaction, the Glaser reaction in the liquid phase was also performed by using a nucleoside analogue **1**; this resulted in the formation of a dimeric nucleoside analogue **2**, as shown in Scheme 2. The synthesis of **H1** was performed in four steps:

1. The incorporation of a reactive alkyne-modified deoxynucleotide into DNA was performed by using the phosphoramidite method.
2. After the first elongation, the support was removed from the synthesizer, and a coupling reaction between two reactive alkynes (an oligonucleotide on the support and a modified monomeric nucleotide in solution) was performed by using a solid-phase Glaser reaction (Scheme 1, step A).
3. The support was washed and reinstalled on the synthesizer, the solid-phase synthesis was resumed and additional DNA bases were introduced (Scheme 1, step B). Upon completion of the second elongation, the support was removed from the synthesizer. The acetylation of two hydroxyl groups at the terminal 5′-position of the oligomers was then performed.
4. Deprotection of the 3′-position of uridine was performed. The support was washed and reinstalled on the synthesizer, and a final DNA-chain elongation was performed in the 5′- to 3′-direction by using reverse nucleoside phosphoramidites (3′-DMTr, 5′-phosphoramidite) (19) (Scheme 1, step C).

**Figure 3.**
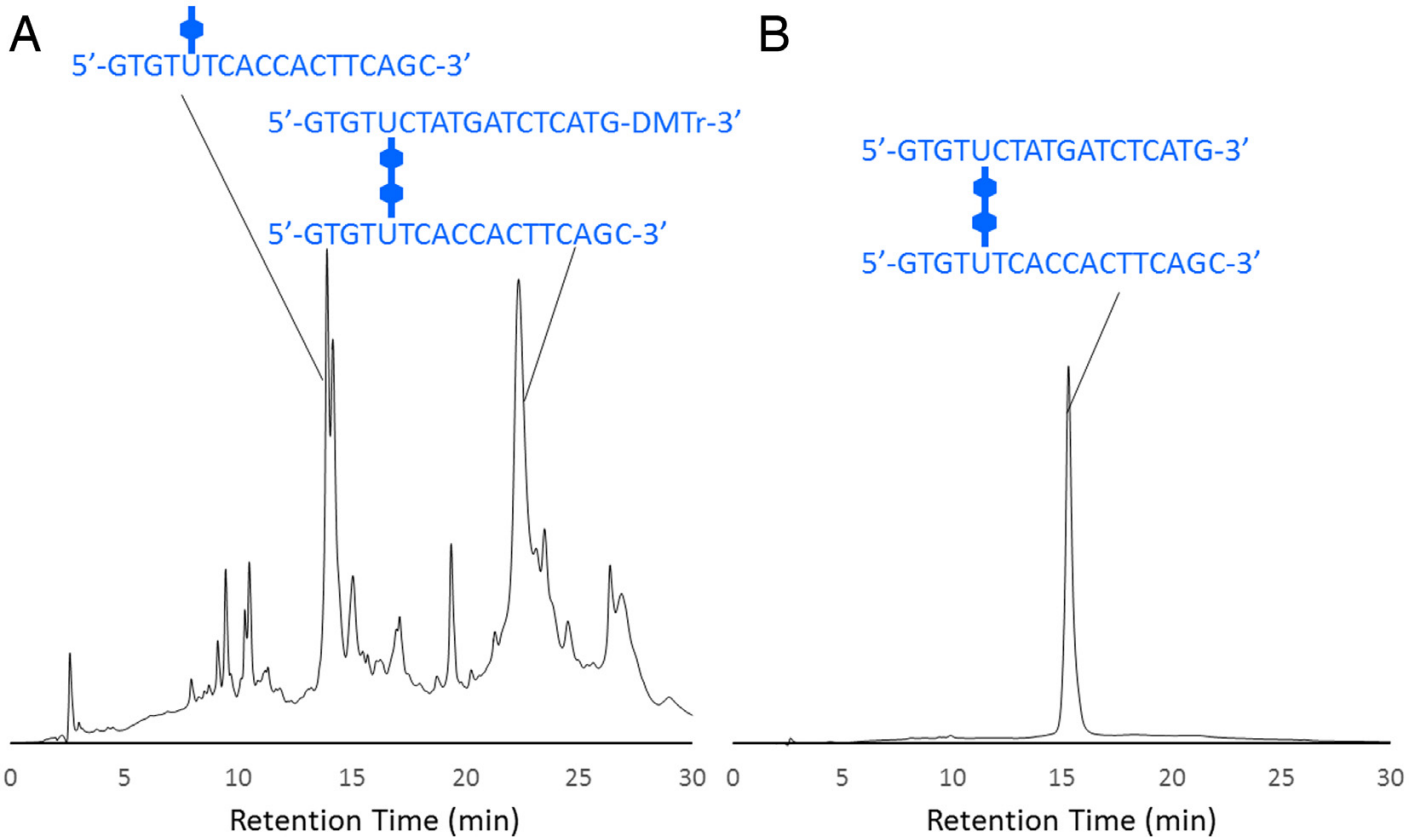
HPLC profiles of the reaction mixture from the synthesis of **H1**. (**A**) Crude reaction mixture obtained on cleaving products from the solid support. (**B**) Product after separation and detritylation. Elution was with 50 mM TEAA containing 3–40% acetonitrile in a linear gradient at a flow rate of 1.0 ml/min for 30 min, at 40°C.

**Figure 4.**
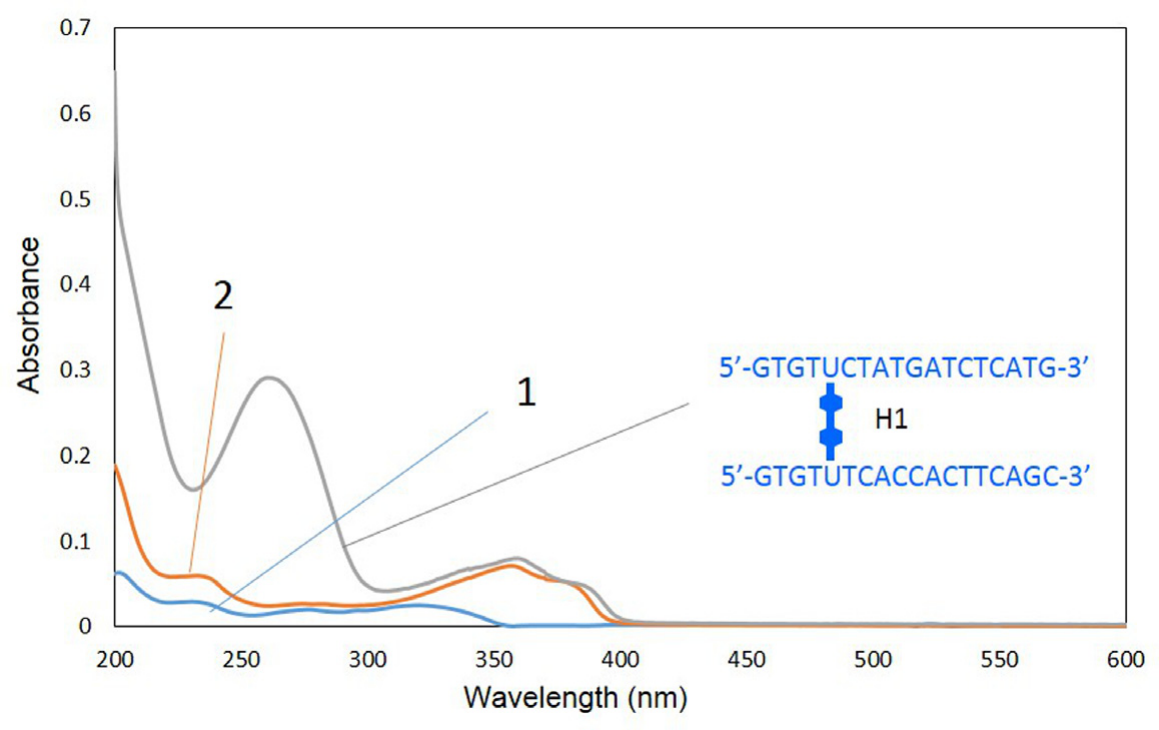
UV-vis spectra of **H1** (10 μM strand concentration, 2 mM sodium cacodylate buffer, pH 7.0), **1** and **2** (10 μM of each, methanol). The spectra were obtained at room temperature using a 1 mm path-length cuvette.

Upon completion of the synthesis, the 3′-DMTr-containing **H1** DNA was cleaved from the support and isolated from the crude reaction mixture by reverse-phase HPLC; the HPLC traces of the crude oligonucleotide products are presented in Figure 3A. By using mass spectrometric analysis, the isolated sample obtained from the distinct peak observed after 22 min was identified as the DMTr-containing **H1** (ESI-TOF MS calcd. for C_390_H_462_N_120_O_222_P_34_ [M-6H]^6−^ 1904.99, found 1905.08). After collecting this fraction and removing the DMTr group from the 3′-end by using acetic acid, the desired unit **H1** was obtained (Figure 3B). **H1** was confirmed by mass spectrometric analysis (ESI-TOF MS calcd. for C_369_H_444_N_120_O_220_P_34_ [M-6H]^6−^ 1854.64, found 1854.95). The sample that eluted at a lower retention time (ca. 14 min) was identified as an elongated oligomer that did not undergo the coupling reaction (Figure 3A). The yield of the linker formation in step a shown in Scheme 1 was 47%, which was sufficient for our experiments. Because the yield is directly linked to the efficiency of the H-shaped DNA formation, improvement of this step is important for the efficient synthesis. For example, coupling yield should be improved by repeating this coupling reaction or increasing the concentration of monomer. **H2** was synthesized and purified in a manner analogous to that of **H1**. Figure 4 depicts the UV-vis spectrum of **H1** compared with the spectra of nucleosides **1** and **2**.

The spectra of **H1** and nucleosides **2** have very similar profiles in the range of 350–400 nm, which indicates that **H1** contains the dimer. The formation of the dimer was also confirmed by comparison of the HPLC analysis of the product from enzymatic digestion of **H1** with that of deprotected **2** (Supplementary Figure S1).

**Figure 5.**
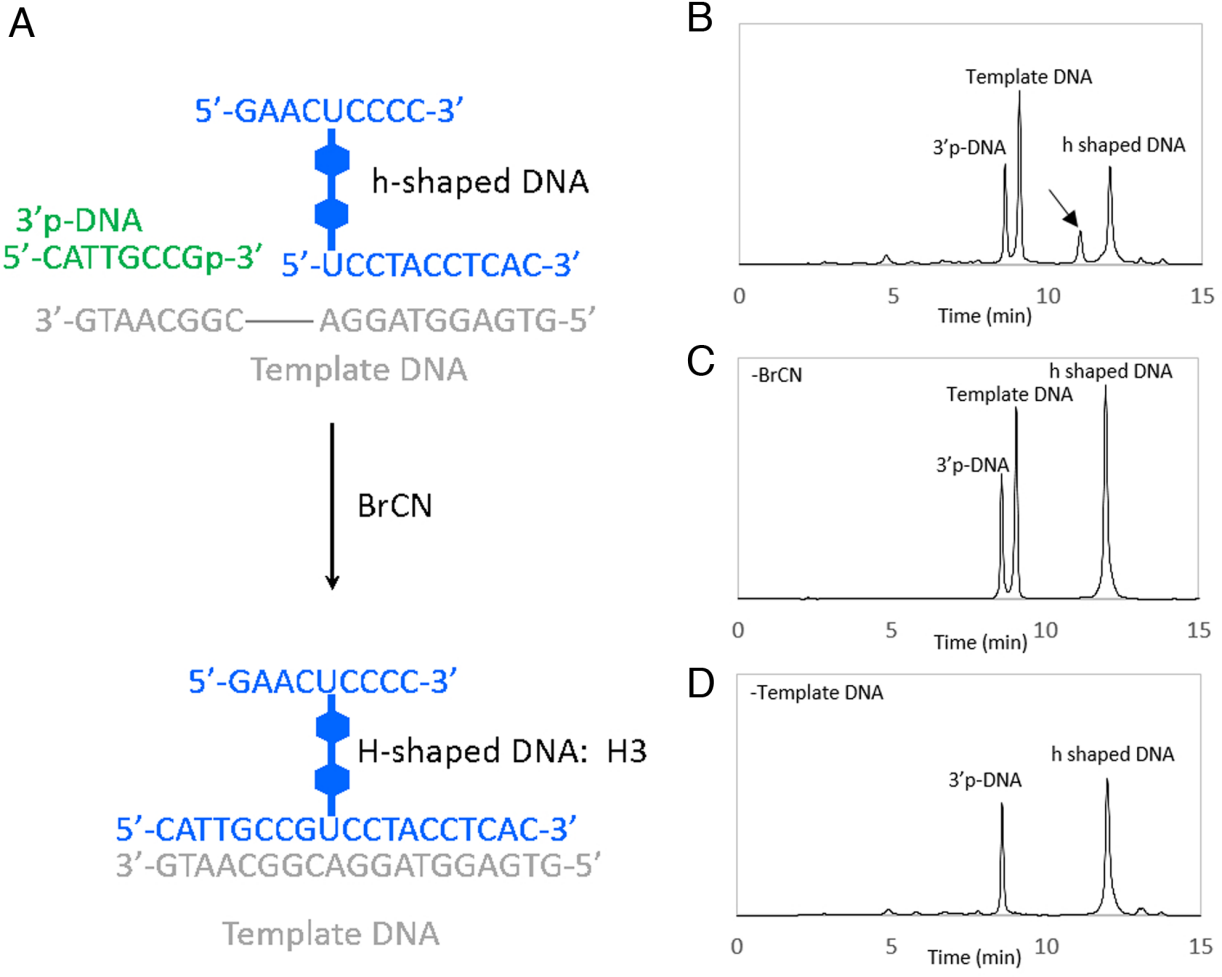
H-shaped DNA synthesis by using chemical ligation. (**A**) Oligonucleotides used in this study. (**B**) HPLC profile of reaction products produced from H-shaped DNA and 3′p-DNA after incubation with template DNA and BrCN. (**C**) Same reaction as (A) but without BrCN. (**D**) Same reaction as (A) but without template DNA. Elution was with 50 mM TEAA containing 3–40% acetonitrile in a linear gradient at a flow rate of 1.0 ml/min for 15 min, at 40°C. HPLC peaks were detected at 280 nm.

**Scheme 3.**
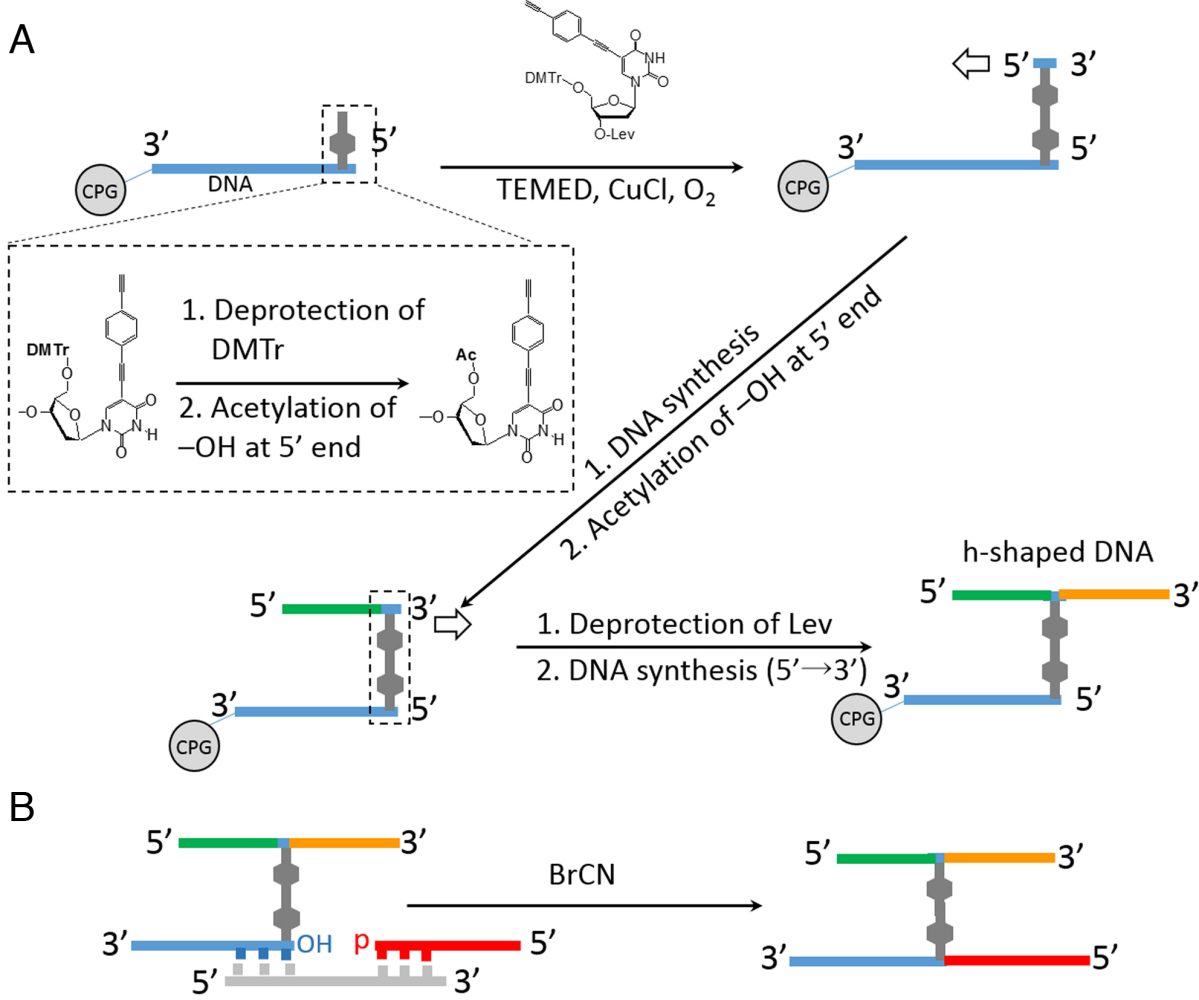
Synthetic procedure of H-shaped DNA which contains non-overlapping sequence. (**A**) On-support synthesis of the H-shaped DNA system. (**B**) Synthesis of H-shaped DNA using chemical ligation.

In the synthetic procedure of H-shaped DNA shown in Scheme 1, DNA sequences elongated after Glaser coupling reaction (shown in green strand) overlap each other. This problem could be solved by combining DNA ligation method with our synthetic procedure. The modified synthetic procedure for the preparation of H-shaped DNA composed of different DNA strands is described below. In the first step, H-shaped DNA, an incomplete structure of H-shape, was obtained by slightly modifying the H-shaped DNA synthesis method as shown in Scheme 3A. After H-shaped DNA was cleaved from a support and purified with HPLC, the H-shaped DNA was converted into an H-shaped DNA by linking with a 3′-phosphoryl DNA oligomer (Scheme 3B). Because chemical structure of the modified region in H-shaped DNA differs from native thymine base, we used non-enzymatic chemical ligation method. According to previous report (20), cyanogen bromide (BrCN) was used as a coupling regent for the phosphodiester bond formation between 5′-hydroxy group at modified region of H-shaped DNA and 3′-phosphoryl DNA (detailed conditions are shown in the Supplementary Data). HPLC profile of the reaction mixture which contains h-DNA, 3′-phosphoryl DNA and template DNA after incubation with BrCN in MES-TEA buffer (pH 7.5) for 10 min is shown in Figure 5B. By using mass spectrometric analysis, the isolated sample obtained from the distinct peak, which was observed after 11 min (indicated by arrow in Figure 5B), was identified as the **H3** (ESI-TOF MS calcd. for C_284_H_346_N_94_O_165_P_26_ [M-5H]^5−^ 1702.50, found 1702.48). This product was not formed after incubation without template DNA (Figure 5D). These results indicate that this product was not formed by the sequence independent random ligation. The combined synthetic procedure will be important when H-shaped DNA contains non-overlapping sequence which is needed for more complicated DNA nanostructure design.

### Construction of DNA units from H-shaped DNA systems

**Figure 6.**
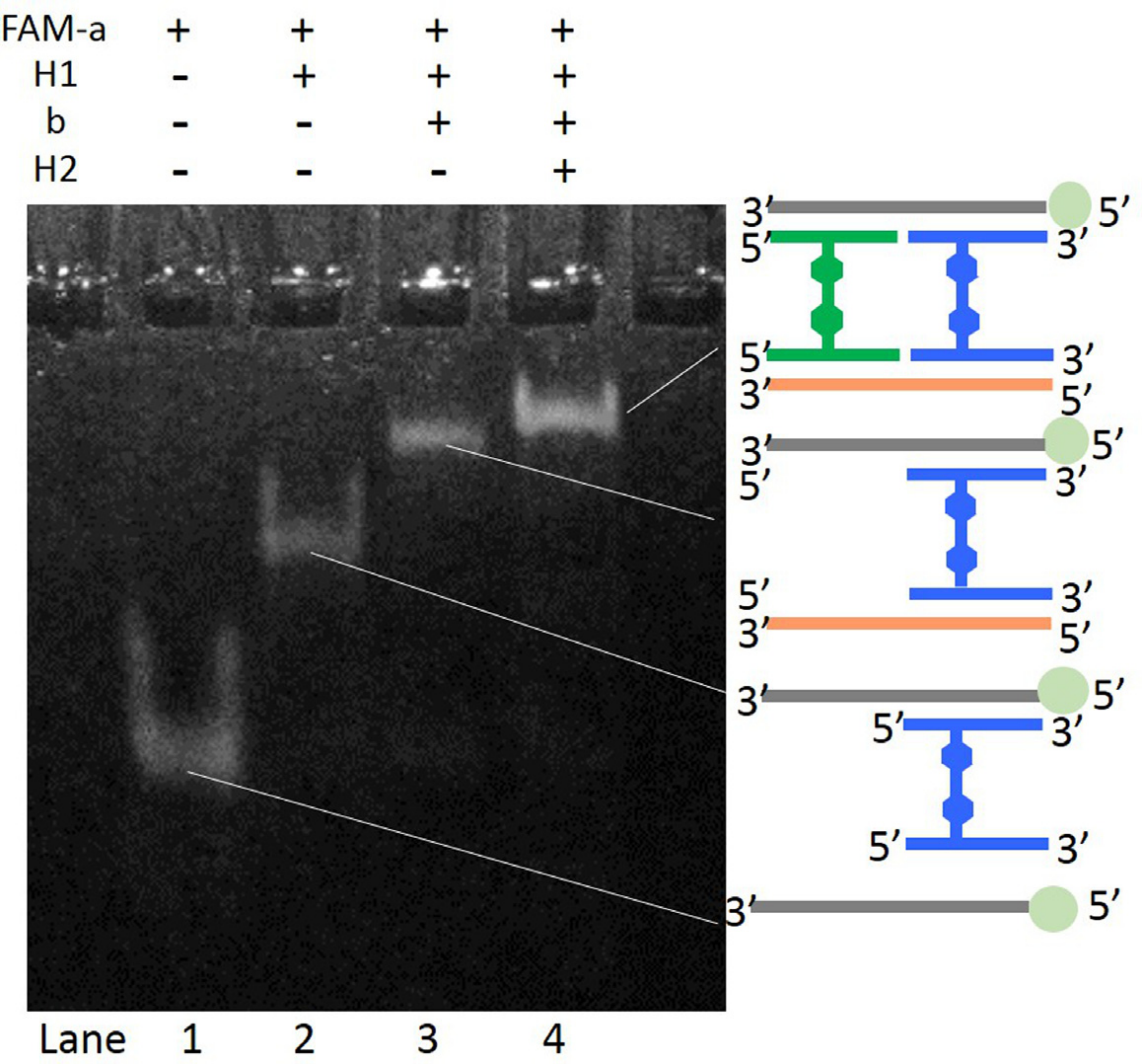
Native PAGE analysis (12.5%) of DNA complexes by sequentially adding DNA building blocks for the formation of **RDU** and **LDU**. The mobile bands were observed by fluorescence of FAM labeled at the 5′-end of **a**. The lanes are as follows: lane 1, **a**; lane 2, **a** + **H1**; lane 3, **a** + **H1** + **b**; lane 4, **a** + **H1** + **b** + **H2**. Conditions: 50 nM of each oligonucleotide, 100 mM NaCl, 10 mM MgCl_2_, 1 × TBE buffer. FAM is represented as a green sphere in the illustrations.

**Figure 7.**
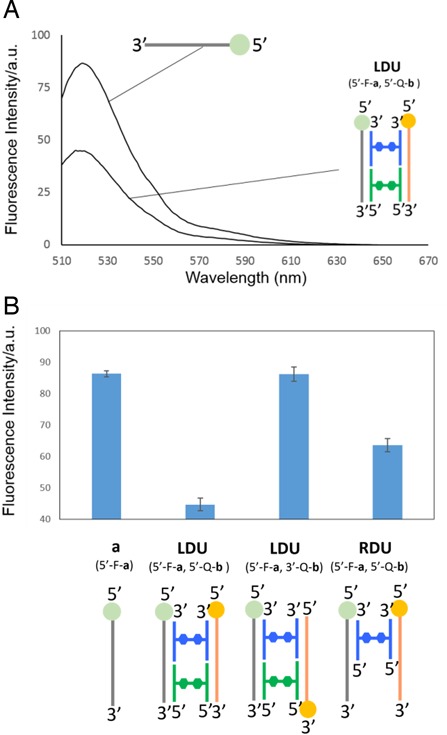
(**A**) Emission spectra of FAM labeled **a** and **LDR** (5′-F-**a**, 5′-Q-**b**). (**B**) Fluorescence intensities of DNA complexes at 520 nm. Conditions: 10 μM of each oligonucleotide, 10 mM NaCl, 10 mM MgCl_2_, 2 mM sodium cacodylate buffer, pH 7.0. The excitation wavelength was 480 nm. The spectra were obtained at 10°C. In the illustrations, FAM (fluorophore) and Dabcyl (quencher) are represented as green and orange spheres, respectively.

We then tested the hybridization of synthesized H-shaped DNA systems with complementary DNA oligonucleotides. Native PAGE analysis was used to confirm the formation of **RDU** and **LDU**. Bands were visualized by the fluorescence of fluorescein (FAM) attached at the 5′-end of **a**. The different mobility of bands observed in lanes 1, 2 and 3 in Figure 6 clearly indicated that the building block **H1** was hybridized with both **a** and **b**, resulting in the formation of the desired **RDU**. The formation of **LDU** by the addition of **H2** was evidenced by the observed band with slightly lower mobility (lane 4). This difference in band mobility was simply because of the difference in the molecular weight of the DNA complexes. These results clearly indicate that the H-shaped DNA system was able to hybridize with complementary DNA, forming the desired **RDU** and **LDU**. To further investigate the formation of the **RDU** and **LDU**, a fluorescent resonance energy transfer experiment was conducted by adding FAM to **a** (represented as 5′-F-**a**) as a fluorophore and 4-(4′-dimethylaminophenylazo)benzoic acid (Dabcyl) as a quencher to the 5′- or 3′-terminal of **b** (represented as 5′-Q-**b** or 3′-Q-**b**) in each complex, respectively (Figure 7). As presented in Figure 6A, fluorescence quenching of FAM by Dabcyl was observed in the **LDU** (5′-F-**a**, 5′-Q-**b**) complex. Conversely, almost no quenching was observed in the **LDU** (5′-F-**a**, 3′-Q-**b**) (Figure 7B). These results indicated the formation of **LDU** and suggested that the two DNA duplexes of this complex were arranged in parallel, resulting in the closing of the 5′-terminal of **a** and **b**. The modest quenching observed for the **RDU** supports this conclusion.

**Figure 8.**
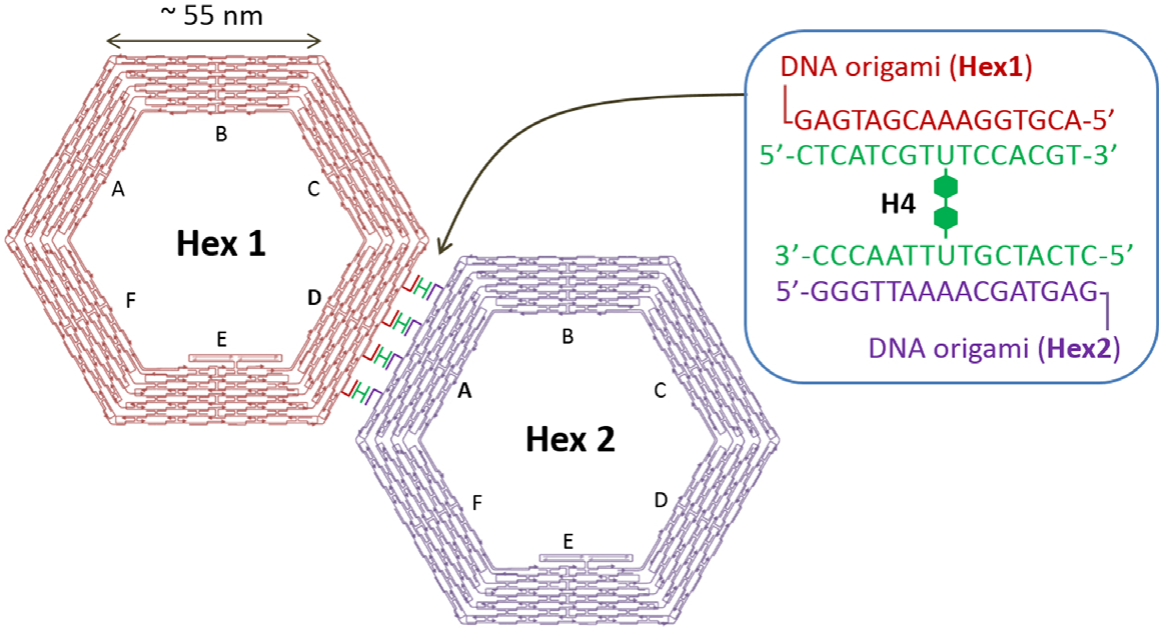
Schematic representation of hexagonal DNA origami dimer connected by H-shaped DNAs (**H4** linker). Four connection strands complementary to the **H4** linker were introduced to the D- and A-edge of the hexagon origami monomers, **Hex1** and **Hex2**, respectively.

**Figure 9.**
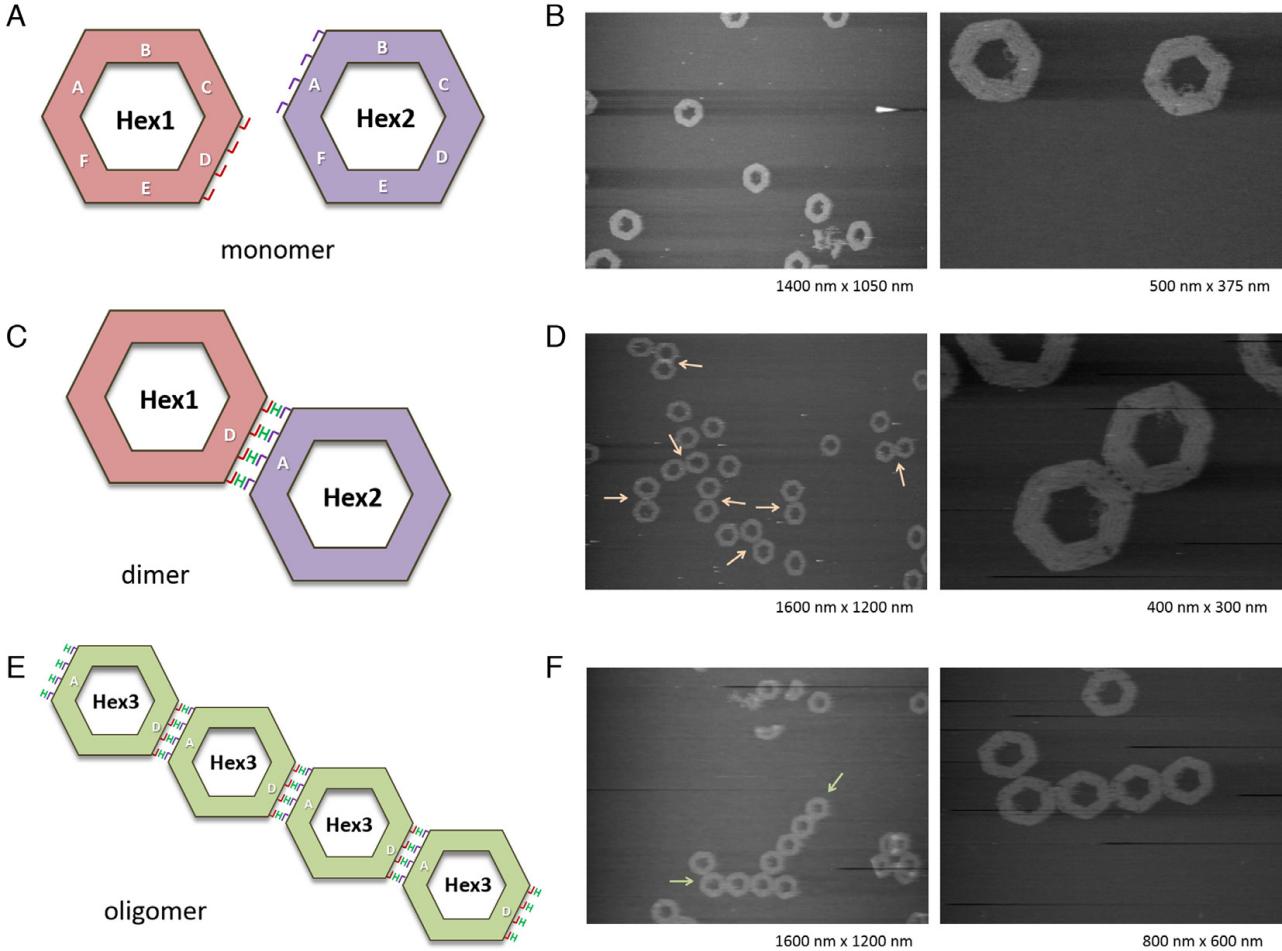
Formation of hexagon origami assemblies connected using H-shaped DNA. (**A**) Hexagon origami monomers, **Hex1** and **Hex2**, having four connection strands at D- and A-edge, respectively. (**B**) AFM images of the mixture of **Hex1** and **Hex2** monomers. (**C**) Hexagon origami dimer formation using **H4** linker with **Hex1** and **Hex2** monomers. (**D**) AFM images of hexagon dimers. Arrows indicate the dimer. (**E**) Hexagon origami oligomers assembled using **H4** linker with **Hex3** monomer which has connection strands at both A- and D-edges. (**F**) AFM images of hexagon oligomers. Arrows indicate the oligomers.

We also investigated the ability of H-shaped DNA to connect 100 nm-sized nanostructures such as hexagonal DNA origami. We designed hexagonal DNA origami having four connection strands complementary to the H-shaped DNA (**H4**) on one edge of hexagonal structure (**Hex1** and **Hex2**) as shown in Figure 8. In this design, **H4** is expected to be a linker unit which connects **Hex1** and **Hex2**. First, hexagonal monomers were designed and constructed using DNA origami technique (Figure 8, Supplementary Figures S2 and S3). After annealing the DNA mixture, hexagonal monomers were observed using AFM. In this condition, no dimer was formed in the mixture of **Hex1** and **Hex2** due to the absence of **H4** linker (Figure 9A and B). Next, **H4** linker was added to the mixture of **Hex1** and **Hex2**, and then the mixture was annealed to form the dimer assembly (Figure 9C, Supplementary Figure S4a). As shown in the AFM images of Figure 9D, hexagonal dimer was formed by hybridization of **H4** linker to the complementary connectors of **Hex1** and **Hex2**. Interestingly, four strands between **Hex1** and **Hex2** were clearly observed, in which **H4** linker and connection strands hybridized to form the dimer. We obtained 50% yield of dimer formation of **Hex1** and **Hex2** by annealing with 6–8 equivalent of **H4** linker.

Furthermore, we designed hexagonal origami which contains connection strands on two edges of a hexagonal structure to form oligomers in the presence of **H4** linker (Figure 9E). As expected, oligomerized hexagonal origami were observed by annealing of **Hex3** monomer with **H4** linker (Figure 9F, Supplementary Figure S4b). These results indicate that H-shaped DNA is capable to connect DNA origami monomers and utilized as a key molecule for various DNA nanostructure construction.

In conclusion, we have designed and synthesized a novel cross-linked DNA system (H-shaped DNA) by connecting two DNA strands through a rigid linker. The H-shaped DNA system does not contain a flexible region between the linker and the two DNA duplexes. By using this framework, a **RDU** was constructed as a first example of a rigid movable segment. In addition to the **RDU**, a **LDU** was also constructed that contained two H-shaped DNA structures. The latter **LDU** motif may also be used as a building block for DNA tiling. Our next challenge will be the construction of large DNA machines containing these units. We will also use real-time AFM to observe the motions of these machines directly (11,21–23). We anticipate that the rigid joint between the two DNA duplexes will constitute a useful movable segment for DNA machines that should allow precise control of motion. Construction of DNA trimer or more complicated unit by conjugation of several DNA strands with multiple linkers will also be our next challenge. Because yield for linker formation is not so high, we need to improve condition of coupling reaction to synthesize such complicated units.

## Supplementary Material

SUPPLEMENTARY DATA
