# Supplementary material for: Linking two DNA duplexes with a rigid linker for DNA nanotechnology
Source: Nucleic Acids Res. 2015 Jun 30;43(14):6692–700. doi: 10.1093/nar/gkv662 (PMC4538841; doi:10.1093/nar/gkv662)
Supplement: SUPPLEMENTARY DATA [file supp_43_14_6692__index.html]

Linking two DNA duplexes with a rigid linker for DNA nanotechnology — SUPPLEMENTARY DATA 

# Linking two DNA duplexes with a rigid linker for DNA nanotechnology

## SUPPLEMENTARY DATA

- SUPPLEMENTARY DATA
