# Supplementary material for: Linking two DNA duplexes with a rigid linker for DNA nanotechnology
Source: Nucleic Acids Res. 2015 Jun 30;43(14):6692–700. doi: 10.1093/nar/gkv662 (PMC4538841; doi:10.1093/nar/gkv662)
Supplement: SUPPLEMENTARY DATA [file supp_gkv662_nar-00969-f-2015-File014.pdf]

SUPPLEMENTARY DATA

Linking Two DNA Duplexes with a Rigid Linker for DNA nanotechnology

Ryu Tashiro[1,*] , Masahiro Iwamoto[1], Hironobu Morinaga[2], Tomoko Emura[2], Kumi Hidaka[2], Masayuki Endo[3,*] and Hiroshi Sugiyama[2,3,*]

[1] Faculty of Pharmaceutical Sciences, Suzuka University of Medical Science, 3500-3 Minamitamagaki-cho, Suzuka-shi, Mie 513-8670, Japan.

[2] Department of Chemistry, Graduate School of Science, Kyoto University, Kitashirakawa-Oiwakecho, Sakyo-ku , Kyoto, 606-8502, Japan.

[3] Institute for Integrated Cell-Material Sciences (WPI-iCeMS), Kyoto University, Yoshida-ushnomiyacho, Sakyo-ku , Kyoto, 606-8501, Japan.

Abbreviations: Ac = acetyl group, CPG = controlled pore glass, BrCN = cyanogen bromide, DMAP = *N,N*-dimethyl-4-aminopyridine, DMF = *N,N* -dimethylformamide, DMSO = dimethyl sulfoxide, DMTr = 4,4'-dimethoxytrityl group, EDTA = ethylenediaminetetraacetic acid, Lev = levulinic group, MES = morpholinoethanesulfonic acid, TBAF = tetrabuthylammonium fluoride, TBE = tris-borate-EDTA, TEA = triethylamine, TEAA = triethylammonium acetate, TEMED = *N,N,N* ',*N* '-tetramethylethylenediamine), THF = tetrahydrofuran, TIPS = triisopropylsilyl group.

Chemical Synthesis

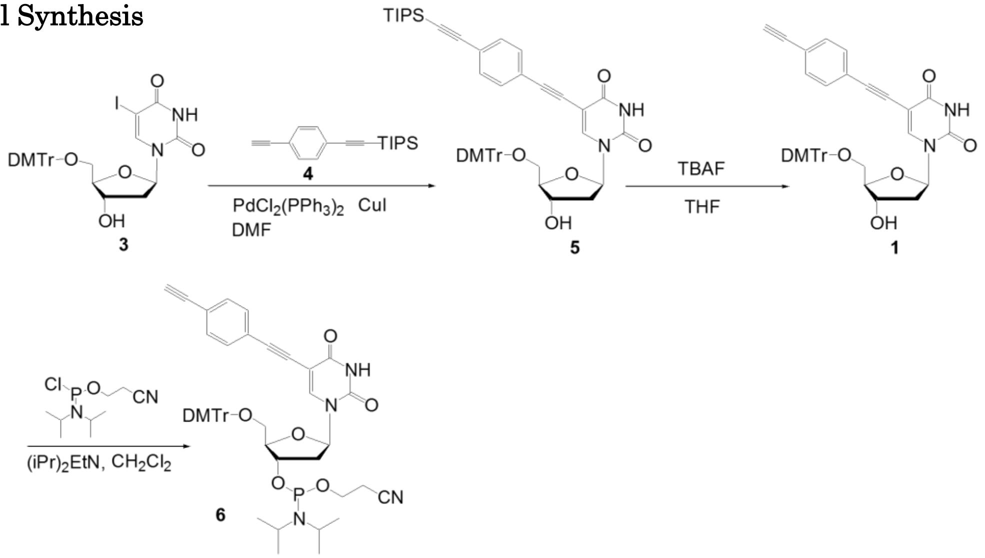

Scheme S1

Compound 5. $PdCl_2(PPh_3)_2$ (21 mg, 0.03 mmol), CuI (5.7 mg, 0.03 mmol) and TEA (0.5 mL) were added to a solution of **3** (200 mg, 0.30 mmol) and **4**[1] (170 mg, 0.60 mmol) in DMF (1 mL). The mixture was degassed and stirred for 3 h under N_2 atmosphere at 50 °C. The mixture was extracted with CH_2Cl_2. The organic phase was dried over Na_2SO_4, filtered, and evaporated to dryness. The residue was purified by flash chromatography on silica gel, eluting with CH_2Cl_2/CH_3OH (v/v 99/1) to afford **5** (174 mg, 0.215 mmol, 66.2%) as a slightly yellow foam. ^{1}H-NMR (400 MHz, $CDCl_3$): δ 8.56 (br, 1H), 8.21 (s, 1H), 7.43 (d, *J* = 7.2 Hz, 2H) 7.34 (d, *J* = 7.4 Hz, 4H), 7.26-7.23 (m, 4H), 7.16 (t, *J* = 7.2 Hz, 1H), 6.94 (d, *J* = 7.2 Hz, 2H), 6.80-6.77 (m, 4H), 6.36 (dd, *J* = 7.2 and 5.6 Hz, 1H), 4.57 (m, 1H), 4.12 (m, 1H), 3.71 (s, 3H), 3.70 (s, 3H), 3.47 (dd, *J* = 11.2 and 3.6 Hz, 1H), 3.39 (dd, *J* = 11.2 and 3.6 Hz, 1H), 2.56-2.52 (m, 1H), 2.51-2.31 (m, 1H), 1.17 (s, 21H) ppm; ^{13}C NMR (100 MHz, $CDCl_3$): δ 161.04, 158.63, 149.06, 144.37, 142.30, 135.43, 135.40, 131.53, 131.38, 129.94, 129.91, 128.08, 127.89, 127.09, 123.00, 122.24, 113.35, 106.64, 100.43, 93.50, 92.72, 87.15, 86.71, 85.88, 81.60, 72.46, 63.45, 55.18, 41.71, 18.66, 11.29 ppm. HRMS (positive): calcd. for $C_{49}H_{55}N_2O_7Si$ $[M+H]^+$: 811.3781, found 811.3779

Compound 1. TBAF (1 M in THF) (150 μL, 0.15 mmol) was added to a solution of **5** (100 mg, 0.12 mmol) in dry THF (5 mL) at room temperature, and the mixture was stirred for 1 h. The solution was evaporated under reduced pressure, diluted with CH_2Cl_2, and washed

with brine. The organic layer was dried on Na_2SO_4, filtered, and evaporated to dryness. The residue was purified by flash chromatography on silica gel, eluting with CH_2Cl_2/CH_3OH (v/v 99/1) to afford **1** (71 mg, 0.11 mmol, 91.7%) as a slightly yellow foam. ^{1}H-NMR (600 MHz, $CDCl_3$): δ 9.11 (s, 1H), 8.25 (s, 1H), 7.43 (d, *J* = 7.2 Hz, 2H), 7.34 (d, *J* = 8.4 Hz, 4H), 7.26-7.23 (m, 4H), 7.14 (t, *J* = 7.5 Hz, 1H), 6.90 (d, *J* = 8.4 Hz, 2H), 6.80-6.76 (m, 4H), 6.37 (dd, *J* = 7.2 and 6 Hz, 1H), 4.57 (m, 1H), 4.14 (m, 1H), 3.69 (s, 3H), 3.68 (s, 3H), 3.48 (dd, *J* = 10.8 and 2.4 Hz, 1H), 3.30 (dd, *J* = 10.8 and 2.4 Hz, 1H), 3.13 (s, 1H), 2.57-2.54 (m, 1H), 2.36-2.31 (m, 1H) ppm; ^{13}C NMR (125 MHz, $CDCl_3$): δ 161.27, 158.61, 149.24, 144.38, 142.49, 135.44, 131.60, 131.45, 129.93, 129.89, 129.14, 128.07, 127.89, 127.07, 122.86, 121.77, 113.35, 113.16, 100.37, 93.26, 87.12, 86.80, 85.92, 83.23, 81.84, 78.87, 72.42, 63.45, 55.18, 41.74 ppm. HRMS (negative): calcd. for $C_{40}H_{33}N_2O_7$ $[M-H]^-$: 653.2288, found 653.2296.

Compound 6. $(iPr)_2EtN$ (140 μL, 0.80 mmol) and 2-cyanoethyl *N,N*-diisopropyl chlorophosphoramidite (89 μL, 0.4 mmol) were added to a solution of **1** (200 mg, 0.31 mmol) in dry CH_2Cl_2 (2 mL). The solution was stirred at room temperature for 30 min. The solution was diluted with CH_2Cl_2 and washed with brine. The organic phase was dried over Na_2SO_4, filtered, and evaporated to dryness. The residue was purified by flash chromatography on silica gel, eluting with AcOEt/Hexane (v/v 2/1) to afford **6** (205 mg, 0.24 mmol, 77.4%) as a slightly yellow foam. ^{1}H-NMR (400 MHz, $CDCl_3$): δ 8.31 and 8.26 (2s, 1H), 7.46-7.13 (m, 11H), 6.88-6.82 (m, 2H), 6.80-6.76 (m, 4H), 6.34 (m, 1H), 4.64 (1H,m) 4.25 and 4.15(m, 1H), 3.70-3.69 (m, 6H), 3.67-3.46 (m, 5H), 3.30-3.27 (m, 1H), 3.13 and 3.13 (2s, 1H), 2.69-2.56 (m, 2H), 2.45-2.42 (m, 1H), 2.41-2.34 (m, 1H) 1.18-1.06 (m, 12H) ppm; ^{31}P NMR (243 MHz, $CDCl_3$) δ 148.96, 148.51 ppm. HRMS (positive): calcd. for $C_{49}H_{51}N_4O_8PNa$ $[M+Na]^+$: 877.3343, found 877.3342.

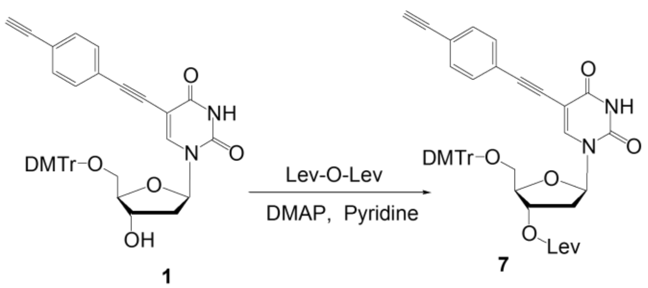

Scheme S2

Compound 7. Levulinic anhydride (43 mg, 0.20 mmol) was added to a solution of **1** (100 mg, 0.15 mmol) and DMAP (2 mg, 0.016 mmol) in dry pyridine (1 mL) at room temperature, and the mixture was stirred for 1 h. The solution was evaporated under reduced pressure, diluted with CH_2Cl_2, and washed with brine. The organic layer was dried on Na_2SO_4, filtered, and evaporated to dryness. The residue was purified by flash chromatography on silica gel, eluting with CH_2Cl_2/CH_3OH (v/v 99/1) to afford **7** (108 mg, 0.14 mmol 93%) as a light yellow foam. ^1H NMR (600 MHz, $CDCl_3$): δ 8.47 (s, 1H), 8.24 (s, 1H), 7.43 (d, J = 7.8 Hz, 2H), 7.34 (d, J = 7.8 Hz, 4H), 7.34-7.23 (m, 4H), 7.15 (t, J = 7.8 Hz, 1H), 6.91 (d, J = 7.8 Hz, 2H), 6.79-6.76 (m, 4H), 6.38 (dd, J = 11.4 and 9 Hz, 1H), 5.46-5.45 (m, 1H) , 4.19 (s, 1H), 3.70 (s, 3H), 3.69 (s, 3H), 3.46-3.41 (m, 2H), 3.45 (d, J = 9 Hz, 1H)-3.42 (d, J = 9 Hz, 1H), 3.14 (s, 1H), 2.78-2.75 (m, 2H), 2.63-2.57 (overlapped m, 3H), 2.46-2.42 (m, 1H), 2.20 (s, 3H) ppm; ^{13}C NMR (125 MHz, $CDCl_3$): δ 206.35, 172.17, 160.75, 158.63, 158.65, 148.98, 144.31, 142.20, 135.33, 135.24, 131.59, 131.51, 129.23, 129.88, 128.09, 127.84, 127.09, 122.82, 121.84, 113.39, 100.72, 93.23, 87.35, 85.39, 84.63, 83.24, 81.56, 78.88, 75.43, 63.59, 55.17, 38.77, 37.78, 29.80, 27.91 ppm. HRMS (negative): calcd. for $C_{45}H_{40}N_2O_9$ $[M-H]^-$: 751.2656, found 751.2627.

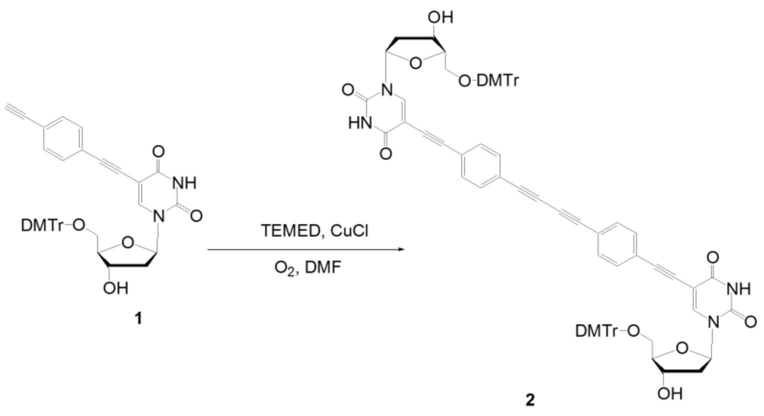

Scheme S3

Compound 2. TEMED (42 μl, 0.28 mmol) was added to a solution of **1** (91 mg, 0.14 mmol) and CuCl (27.2 mg, 0.28 mmol) in DMF (2 mL), and the mixture was stirred for 2 h under O_2 atmosphere at room temperature. The solution was diluted with CH_2Cl_2, and washed with saturated NH_4Cl solution. The organic layer was dried on Na_2SO_4, filtered, and evaporated to dryness. The residue was purified by flash chromatography on silica gel,

eluting with CH_2Cl_2/TEA/CH_3OH (v/v/v 93/5/2) to afford **2** (80.4 mg, 88%) as a white foam. ^{1}H NMR (400 MHz, DMSO-d_6): δ 11.79 (s, 2H), 8.13 (s, 2H), 7.49 (d, J = 8.0 Hz, 4H), 7.41 (d, J = 7.6 Hz, 4H), 7.30-7.25 (m, 12H), 7.15 (t, J = 7.2 Hz, 2H), 7.06 (d, J = 8.0 Hz, 4H), 6.84 (d, J = 3.6 Hz, 4H), 6.82 (d, J = 3.6 Hz, 4H), 6.13 (t, J = 6.4 Hz, 2H), 5.35 (m, 2H), 4.32 (m, 2H), 3.64 (s, 12H), 3.18 (m, 4H), 2.26 (m, 4H) ppm; ^{13}C NMR (100 MHz, DMSO-d_6): δ 161.74, 158.51, 149.73, 145.14, 143.88, 135.98, 135.86, 132.85, 131.76, 130.05, 128.37, 128.05, 127.18, 124.16, 120.38, 113.68, 98.56, 91.82, 86.54, 86.37, 85.64, 82.74, 75.61, 70.80, 63.95, 55.40 ppm. HRMS (negative): Calcd. for $C_{80}H_{65}N_4O_{14}$ [M-H]$^-$: 1305.4497, found 1305.4473

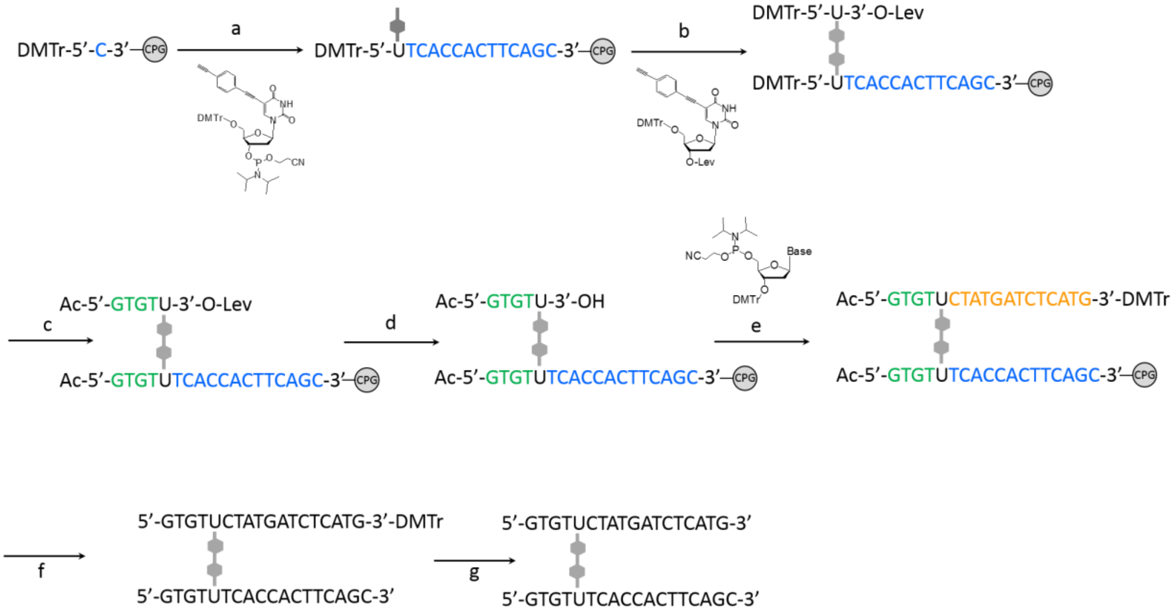

Scheme S4 (a) First DNA synthesis, (b) On-support Glaser reaction, (c) Second DNA synthesis and Acetylation of 5'-end, (d) Removal of Lev protecting group, (e) Third DNA Synthesis, (f) Cleavage of DNA oligomer from CPG, (g) Detrithylation and HPLC purification.

First Automated DNA Synthesis (Scheme S4a). Synthesis of DNA oligomers was carried out on 1μM scale with an AppliedBiosystems 3400 DNA synthesizer. A standard program was used for standard 3' CE (β-cyanoethyl) nucleotide phosphoramidites. When coupling reaction was performed with **6,** coupling time was extended to 3 minutes.

Procedure of On-Support Glaser Reaction (Scheme S4b). The DNA-bearing CPG, **6** (10 mg, 0.013 mmol), CuCl (2.6 mg, 0.026 mmol), DMF (150 μL) and TEMED (3.9 μL, 0.026 mmol) were added into a glass tube. The atmosphere in the glass tube was replaced with O_2. The glass tube was sealed and shaken at room temperature for 15 min. The solution phase was removed from this tube and this reaction process was repeated again. After the second reaction cycle was finished, the CPG was washed with DMF (1 mL x 3), 10 mM NH_4Cl aq (1 mL x 2), 10 mM EDTA aq (1 mL x 2), H_2O (1 mL x 3) and CH_3CN (1mL x 3). Washed CPG was dried under vacuum.

Second Automated DNA Synthesis and Acetylation of 5'-OH (Scheme S4c). Second DNA elongation was performed using a standard program. After second elongation was finished, acetylation of 5'-OH was performed by running a synthetic program in the absence of phosphoramidite. 5'-OH was acetylated through capping process in the synthetic cycle.

Removal of Lev protecting group (Scheme S4d). A reported procedure was used with slight modifications.[2] The DNA-bearing CPG was treated with a solution of 0.5 M $NH_2NH_2 \cdot H_2O$ in Pyridine/Acetic acid (1:1 v/v, 0.5ml) for 10 min in a DNA synthetic column. The CPG washed three times with pyridine/acetic acid (1:1 v/v, 1 ml x 3), three times with acetonitrile (1ml x 3) and dried.

Third Automated DNA Synthesis and Cleavage from CPG (Scheme S4e, f). Third DNA elongation was performed with 5'-CE phosphoramidites (GlenResearch) using a standard program (final trithyl-on mode). After synthesis was finished, oligonucleotide was deprotected in a mixture of saturated solution of NH_4OH aq and $MeNH_2$ aq (1:1 v/v, 1 mL) (20min, 65 °C). The solution was dried down to yield the crude DNA mixture.

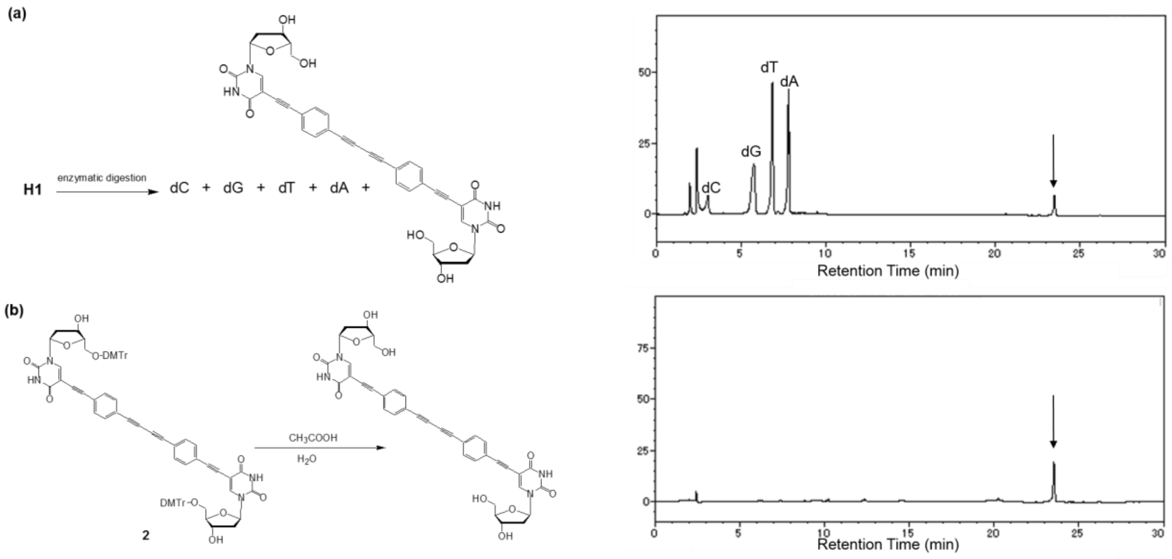

Figure. S1. (a) HPLC profiles of the reaction products obtained after enzymatic digestion of **H1 (top)** by P1 endonuclease and alkaline phosphatase. (b) HPLC profiles of the reaction products obtained after deprotection of **2** with acetic acid/H_2O (4/1). Elution was with 50 mM TEAA containing 0-80% acetonitrile in a linear gradient at a flow rate of 1.0 mL/min for 40 min, at 40 °C.

Procedure of template directed chemical ligation. This reaction was performed according to reported procedure with slight modifications [3]. The h-shaped DNA, 3'-phosphonylated DNA and the template DNA were combined in a 1:1:1 ratio (10 µM each) with $MgCl_2$ (20 mM) in 9 µl MES-TEA buffer (250 mM, pH 7.5). A solution of BrCN (1 µl, 1 M in CH_3CN) was added and incubated for 10 min at 4°C.

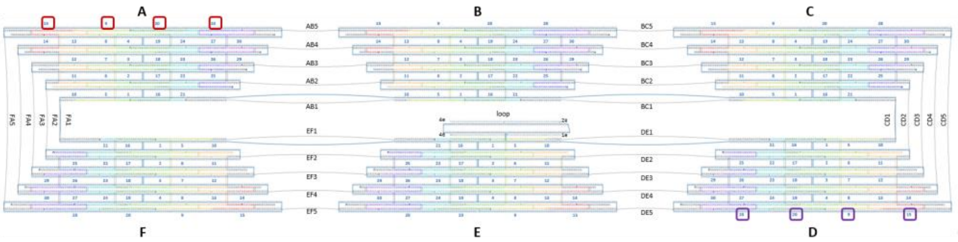

A15-X	X-TGGCTCATTATACCAGTCAGGACGCTAACGGA
A09-X	X-CATTGTGAATTACCTTATGCGATTTTCATCAG
A20-X	X-TGGGCTTGAGATGGTTTAATTTCAACTTTAAT
A28-X	X-CCTGACGAGAAACACCAGAACGAGTCTTGACA

D15-X	X-AGAAACAATAACGGATTCGCCTGAGAGGCGAA
D09-X	X-GAATATACAGTAACAGTACCTTTTAAAGAAGA
D20-X	X-AATTGCGTAGATTTTCAGGTTTAACGTCAGAT
D28-X	X-AAAATTATTTGCACGTAAAACAGACAATTCAT

Figure S2. Hexagon origami design and staple strands in the A- and D-edges for modifications.

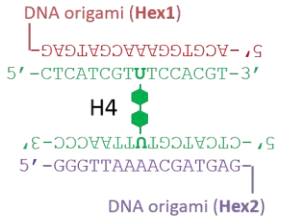

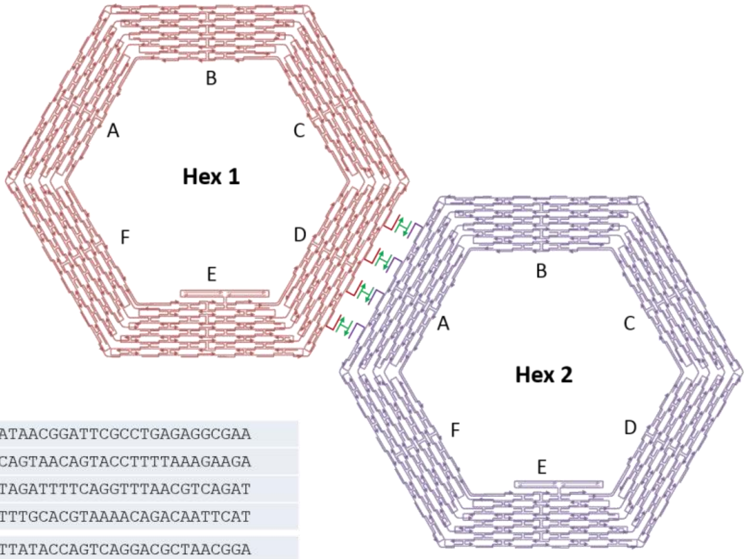

Hex1_D15-H3a′	ACGTGGAAACGATGAG-AGAAACAATAACGGATTCGCCTGAGAGGCGAA
Hex1_D09-H3a′	ACGTGGAAACGATGAG-GAATATACAGTAACAGTACCTTTTAAAGAAGA
Hex1_D20-H3a′	ACGTGGAAACGATGAG-AATTGCGTAGATTTTCAGGTTTAACGTCAGAT
Hex1_D28-H3a′	ACGTGGAAACGATGAG-AAAATTATTTGCACGTAAAACAGACAATTCAT
Hex2_A15-H3b′	GGGTTAAAACGATGAG-TGGCTCATTATACCAGTCAGGACGCTAACGGA
Hex2_A09-H3b′	GGGTTAAAACGATGAG-CATTGTGAATTACCTTATGCGATTTTCATCAG
Hex2_A20-H3b′	GGGTTAAAACGATGAG-TGGGCTTGAGATGGTTTAATTTCAACTTTAAT
Hex2_A28-H3b′	GGGTTAAAACGATGAG-CCTGACGAGAAACACCAGAACGAGTCTTGACA

Figure S3. **H4** linker and connection strands for assembling hexagon origami monomers **Hex1** and **Hex2**.

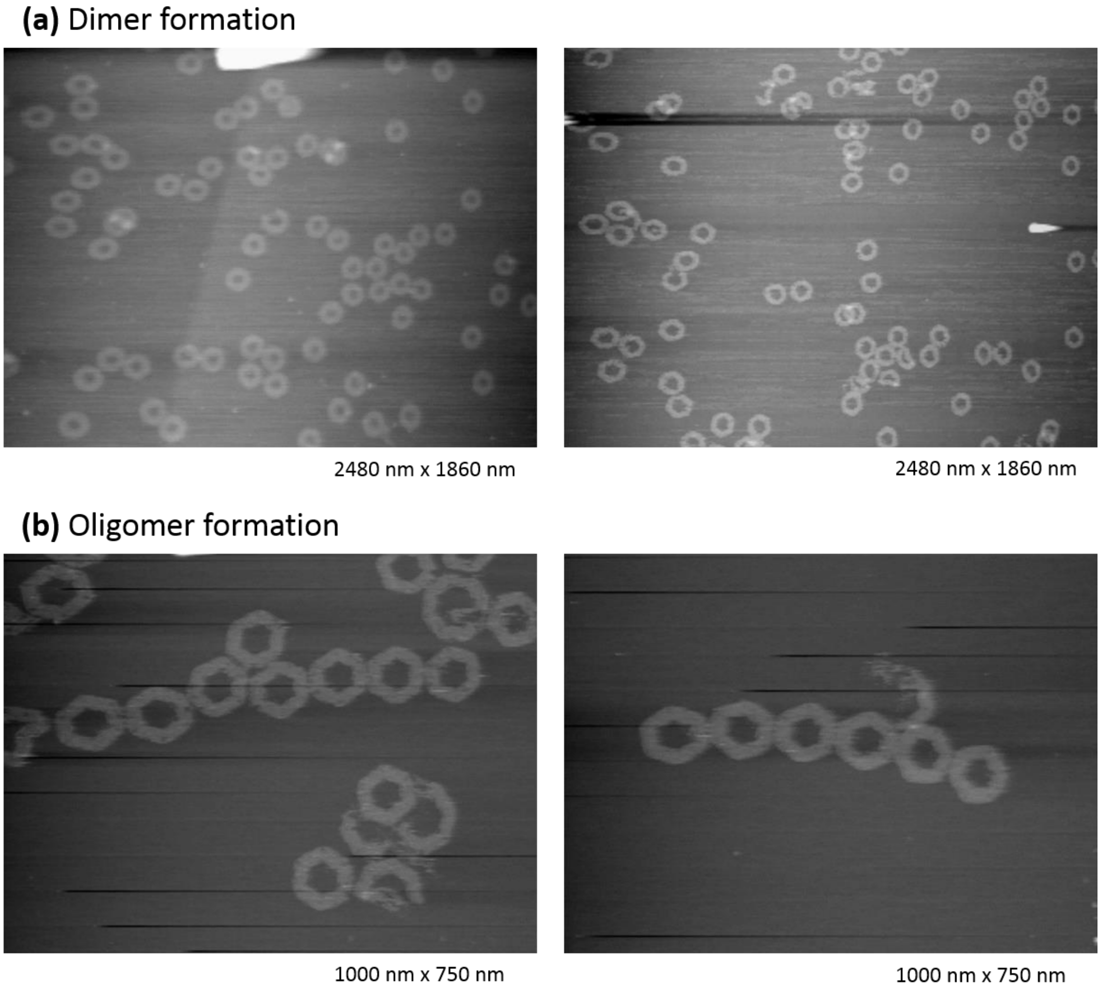

Figure S4. (a) Additional AFM images of **Hex1/Hex2** dimer assembled with **H4** linker. (b) Additional AFM images of **Hex3** oligomer assembled with **H4** linker.

References

[1] J.-F. Nierengarten, S. Zhang, A. Gégout, M. Urbani, N. Armaroli, G. Marconi, Y. Rio *J. Org. Chem.* **2005**, *70*, 7550-7557.

[2] H-Y. Li, Y-L. Qiu, E. Moyroud, Y. Kishi, *Angew. Chem. Int. Ed.* **2001**, *40*, 1433-7851.

[3] N. G. Dolinnaya, N. I.Sokolova, D. T. Ashirbekova, and Z. A. Shabarove. *Nucleic Acids Res.* **1991** *19*, 3067-3072.
